# A Market Basket Survey of Horticultural Fruits for Arsenic and Trace Metal Contamination in Southeast Nigeria and Potential Health Risk Implications

**DOI:** 10.5696/2156-9614-7.15.40

**Published:** 2017-09-07

**Authors:** Chigozie Damian Ezeonyejiaku, Maximilian Obinna Obiakor

**Affiliations:** 1 Department of Zoology, Nnamdi Azikiwe University, PMB 5025, Awka, Anambra State, Nigeria; 2 School of Environmental and Rural Science, University of New England, Armidale, NSW 2351, Australia

**Keywords:** As, Cu, Hg, Pb, food safety, dietary toxicity, public health

## Abstract

**Background.:**

Elevated arsenic and trace metal contamination of the terrestrial food chain represents one of the most significant environmental risk exposures for human populations in developing countries. Metalloid and metal contamination in horticultural crop produce such as fruit is a public health concern in Nigeria. Local fruits are cheap sources of vitamins and minerals for the resident population and pose an important dietary threat of metal(loid) toxicity through consumption.

**Objectives.:**

Market basket investigation of five locally grown (guava, pineapple, orange, and pawpaw) and imported (apple) fruits was conducted to measure the total concentrations of arsenic (As), mercury (Hg), copper (Cu), and lead (Pb) present in these fruits from southeastern Nigeria (Awka, Anambra).

**Methods.:**

Fruits were analyzed for As and the three metals using atomic absorption spectrophotometry. Moisture content of fruits was determined and used to transform metal concentrations in dry weight to wet weight and compared to Codex food grade standards and assorted (sub)tropical fruits, edible and inedible peels.

**Results.:**

The mean ± standard deviation of elemental concentrations in dry weight ranged from 20.0±0.71–96.84±0.00 μg g^−1^ for As, 0.02±0.02 – 0.89±0.33 μg g^−1^ for Hg, 0.11±0.01 - 0.18±0.40 μg g^−1^ for Cu, and <0.001 – 0.03±0.05 μg g^−1^ for Pb. The As concentrations (wet weight) in fruits were ~32–166 orders of magnitude higher than Codex Alimentarius Commission (Codex) maximum As food grade levels. Guava and apple methyl Hg concentrations were ~6–~1 orders of magnitude higher than Codex maximum levels, while the content of Cu and Pb in fruits were within acceptable standard limits.

**Conclusions.:**

The significant concentrations of As and Hg in the examined fruits indicate a potential public health threat. Efforts are needed to initiate and sustain continued monitoring of trace elements in fruits and food sold to consumers due to variation in contaminating sources to ensure food safety. Although a great deal of information exists on Hg toxicity, research on metalloids such as As remains limited in Nigeria and no reliable guidelines exist. Further research is recommended to determine the ecotoxicity of As in Nigeria.

## Introduction

Food systems in developing countries are not always as organized as in developed countries due to problems associated with a burgeoning population, rapid urbanization, and shortfall of resources needed to handle food processing.^1^ In addition, environmental dispersion of contaminants from uncoordinated industrialization and waste management systems further compromise food quality and safety, and raise the probability of dietary risk exposure among consumers.^1–5^ In addition to the fecal and microbial food contamination often related to poor hygiene and sanitation, risks of metal and metalloid (generally referred herein as metals except as otherwise indicated) ingestion through food consumption and associated health implications have received increased attention.^6–14^ Trace metals posing significant health risks (e.g., mercury (Hg), copper (Cu), lead (Pb), and Zinc (Zn)) have been detected in developing countries such as Nigeria, Egypt, Iran, and Bangladesh at substantial concentrations in food crops such as fruits, vegetables and cereals, and have prompted a debate on the efficacy of food quality assurance and safety assessment measures.^15–20^ Although metal contaminants may originate from discrete sources including soil, fertilizers, and atmospheric particulates, accumulation in plants may depend on the species, soil characteristics, metal properties, and environmental conditions.^21–23^ Beyond dietetic metal exposure, air and water are significant random pathways of exposure reported in many case studies.^2–4^,^24^,^25^ The several mutually reinforcing physical and chemical properties of metals nevertheless enhance ease of bioaccumulation and biomagnification in food webs.^26–31^ The growing concern of entrance and transference of metals into terrestrial food webs stems from the potential for adverse effects on the health status of both humans and animals continuously exposed to such harmful contaminants via food consumption.^6^ A number of studies have been conducted on trace metals linked to the incidence of gastrointestinal cancer, and cancer of the pancreas, urinary bladder or prostate.^9^,^32–34^ It is therefore crucial to determine the concentrations of metals present in food as a practical step for effective risk assessment.^6^

While trace metals (often regarded as heavy metals) have been studied extensively and reliable guidelines developed in many jurisdictions, information on metalloid ecotoxicity is relatively limited and not fully understood.^35^ One of the few environmentally and biologically important metalloids, arsenic (As) has been widely studied due to its public health implications and identified multiple pathways of human exposure.^36–39^ The carcinogenicity of As in humans is well known. Inorganic As is a class one human carcinogen whose chronic ingestion may cause cancers of the bladder, kidney, liver, lung, prostate and skin.^40^,^41^ Environmental pollution resulting from industrial activities such as mining and smelting and production processes can increase the dispersion of As and contamination of agronomic crops through water and atmospheric deposition.^20^,^35^,^42–44^ Farm irrigation using As-contaminated groundwater has been shown to elevate As concentrations in rice grains.^45^ Food standards have not been widely developed for As in many countries, although efficient screening exercises and risk communication strategies are in place in most developed countries to safeguard consumers from As exposure.^20^,^46^ No such safeguards are in place in many developing African countries.

**Abbreviations**
*Codex*: Codex Alimentarius Commission
*CRM*: Certified reference materials
*dw*: Dry weight
*ww*: Wet weight

In Nigeria, there are poor food quality controls and existing mechanisms are not very effective at protecting consumers. Deleterious effects of food contaminants have been underreported in the country as a result of inadequate risk assessment, communication and resource deficits.^47^ Contamination of metals in commonly consumed horticultural fruits and effects on human health have not been previously investigated. In this study, we investigated As and trace metals (Hg, Cu, and Pb) contamination in orange (Citrus sinensis), pawpaw (Carica papaya), guava (Psidium guajava), apple (Malus domestica), and pineapple (Ananas comosus) commonly supplied in the Awka region where there has been a history of metal smelting activities.^48^ This market basket survey aimed to collate elemental concentration data and compare to internationally acceptable standards for use in preliminary exposure assessments for the local population.

## Methods

### Location Description and Sample Collection

**Figure 1:**
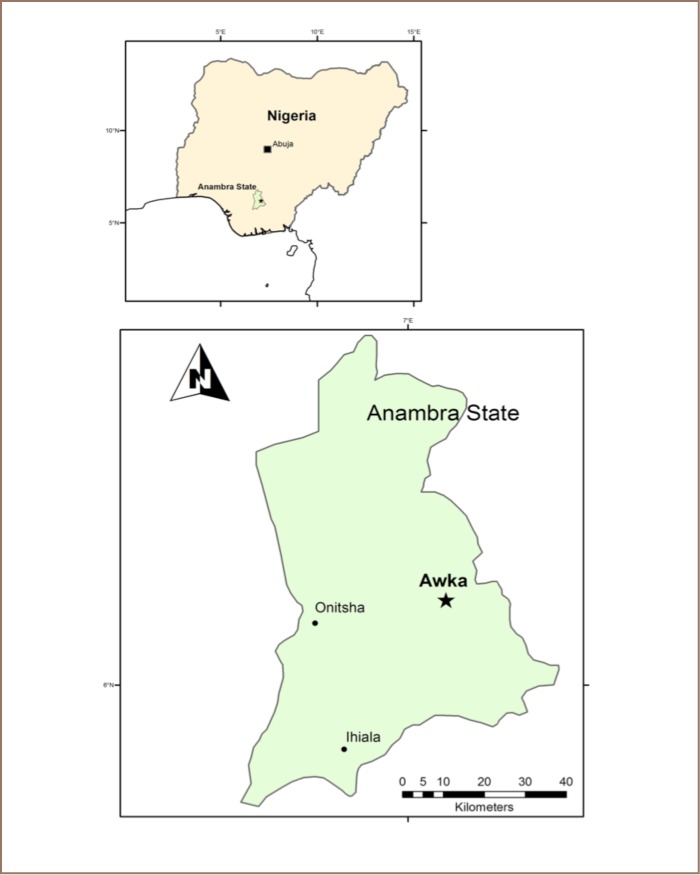
Map of Anambra State and the capital Awka

Samples of locally supplied fruits were collected from the largest market in Awka South, which serves the entire Local Government Area and surrounding towns of Awka North. Awka South is in Anambra State of southeastern Nigeria. It is located within ~6.642° N and ~7.067° E (*Figure 1*) and experiences two distinct seasons brought about by two predominant winds that rule the area: the southwestern monsoon winds from the Atlantic Ocean and the northeastern dry winds from across the Sahara Desert. Seven months of heavy tropical rains (April–October) are followed by 5 months of dryness (November–March). Awka South is generally hot and humid with a temperature range of 27–28°C from July through December but rising to 35°C between February and April.^2^,^49^ It has an estimated population of over 400,000 inhabitants based on 2017 population projection.^50^ The region is comprised of Awka city (capital of Anambra State), Amawbia, Ezinato, Nibo, Nise, Umuawulu, Isiagu, Okpuno, and Mbaukwu. Awka city is the location of many commercial and social facilities and has a history of indigenous technology and crafts, including wood processing, metal smelting and blacksmithing. The uncoordinated waste management systems that characterize agriculture, wood processing and industrial metal activities in the area have led to increased environmental pollution through indiscriminate disposal and dumping of tailings.^48^ Although there has been a gross decline of these metal processing activities due to rapid technological development, unregulated artisanal production still exists.^48^ The city is also one of the most important industrial, economic, cultural, political, and commercial centres in southeast Nigeria. It continues to undergo heavy urbanization and infrastructure development. The industrial facilities in Awka South are scattered at the city centre and surrounding suburbs.

Local fruits were purchased from a wide variety of retailers at the market (known as Eke Awka), including fruits sold openly on market tables and shelves.^20^ All fruits were locally supplied except for apples, which are largely imported in Nigeria from South Africa. Ten samples were purchased for each of the market basket survey fruit (orange, pawpaw, guava, apple, and pineapple). It was not determined if use of adulterants had any effects on apples imported into the country; thus, such effects were deemed to be negligible during preparation and analysis.

### Sample Preparation

Fruit samples were taken to a laboratory in an air-tight container to prevent further atmospheric deposition that might interfere with elemental analysis. Samples were neither washed nor peeled prior to preparation. The intent was to reflect dietary exposures and common consumer food handling strategies, where fruits are purchased from an open market and consumed without washing. Samples were finely diced in a food processor, which was thoroughly cleaned with 10% extra and rinsed in distilled water after processing each sample. Ground samples were then frozen until further processing.

### Analytical Determination of Arsenic, Mercury, Copper, and Lead in Fruit Samples

All samples were analyzed for total As, Hg, Cu, and Pb using established methods with modification.^46^,^51^ Analytical grade reagents were used throughout the metal determination with purity in the range of 98–99.99%. Samples were oven dried at 55°C. Approximately 0.5 g each of the dried fruits was accurately weighed out into 50 ml polyethylene centrifuge tubes; 5 ml of concentrated nitric acid was added to each sample and then left overnight. We digested the samples in a pre-programmed microwave digestion system at a changing temperature and time: 5 minutes ramp to 60°C and then held at 60°C for 10 minutes (400 W); 5 minutes ramp to 75°C and then held at 75°C for 10 minutes (400 W); and 5 minutes ramp to 100°C and then held at 110°C for 30 minutes (400 W). All microwave digestions were carried out in triplicate with a reagent blank. After cooling, digested samples were diluted to 50 ml and total As, Hg, Cu, and Pb analysis was performed by atomic absorption spectrophotometry at a commercial laboratory.

#### Quality Control

Each analytical batch of ten runs was accompanied by an acid blank, and three certified reference materials (CRM): apple leaves, (National Institute of Standards and Technology (NIST) Standard Reference Material 1515); pine needles, (NIST Standard Reference Material 1575a); and rice flour, (NIST Standard Reference Material 1568a) to monitor for instrument accuracy and method extraction efficiency. Mixed element internal standards including all examined metals were measured as part of the analytical quality control. Thus, a total of 25 independent digests including reagent blanks and CRMs were analyzed. Mean recoveries were in an acceptable range (75–98.5%) compared to the CRM theoretical or certified values for the elements. All glassware used in the analyses were cleaned with a detergent-free solution, soaked in acid (10% hydrochloric acid) and then rinsed with metal-free distilled water. Metal concentrations were reported in μg/g dry weight at instrumental detection limit of 0.001 μg/g.

### Moisture Content Determination

We determined the percentage (%) moisture contents of all the fruits examined to enable transformation of metals concentrations from dry weight (dw) to wet weight (ww) basis, and facilitate the comparison of our data with those of Codex Alimentarius Commission (Codex) guideline values (As = 0.50 μg/g ww, Hg = 0.10 μg/g ww, Cu = 2.00 μg/g ww, and Pb = 0.10 μg/g ww). Approximately 10 g of each fruit was weighed into aluminum foil, and dried for 48 hours in a 105°C oven. At the end of the drying period, samples were reweighed and % moisture content was calculated using Equation 1.

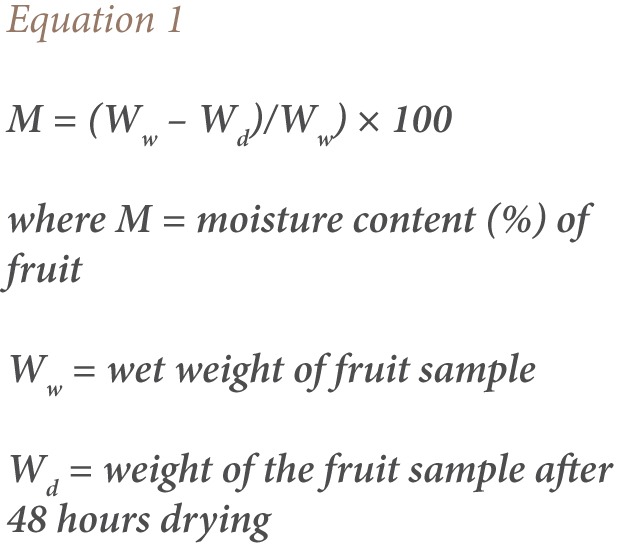



### Statistical Analysis

Data generated from the study were subjected to analysis using IBM SPSS Statistics computer software program (version 22, SPSS Inc., Chicago, IL, USA) and Microsoft Excel (version 2016) at an alpha error of 0.05 and 95% confidence interval. The normality of data distribution was objectively evaluated by the Shapiro-Wilk test, and homogeneity of variance by Levene's test.^52^ We compared the concentrations of the metals in fruits using the Kruskal-Wallis method, a nonparametric analysis of variance test based on rank transformation, with Bonferroni correction and Monte Carlo approximation for unbiased estimate of the exact P value (based on 100,000 random tables from the reference set using a starting seed of 2,000,000).^53^ Metal concentration values less than the maximum detection level were replaced with 0 prior to analysis.

## Results

**Figure 2:**
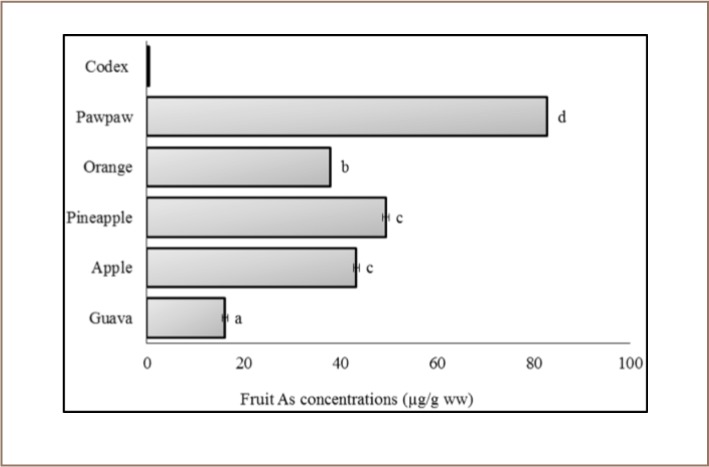
Arsenic concentration of the fruits sampled in the Awka market basket survey. Values represent mean As concentration ±standard deviation, n = 3. Mean values bearing different superscripts are significantly different (Kruskal-Wallis with Bonferroni correction, P < 0.05). Codex indicates Codex food grade standard.^55^

**Figure 3:**
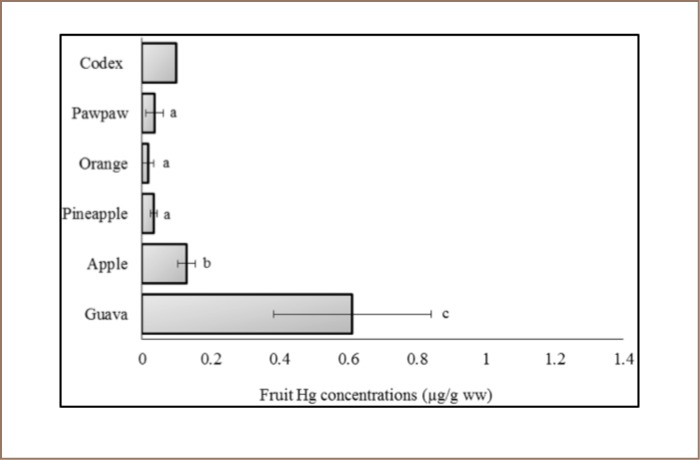
Methyl mercury concentration of the fruits sampled in the Awka market basket survey. Values represent mean methyl Hg concentration ±standard deviation, n = 3. Mean values bearing different superscripts are significantly different (Kruskal Wallis with Bonferroni correction, P < 0.05). Codex indicates Codex food grade standard.^55^

**Figure 4:**
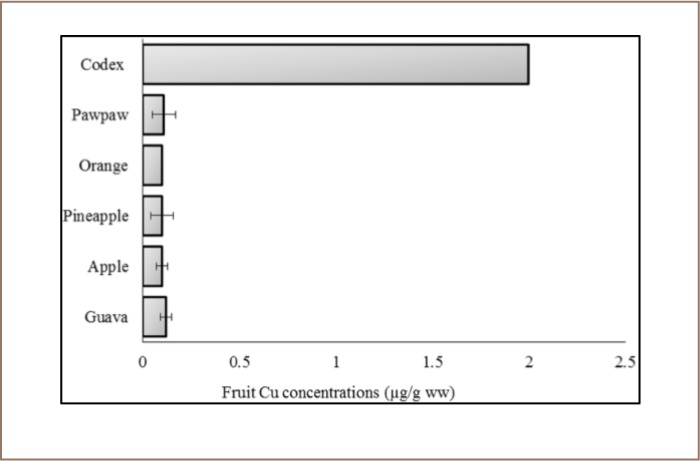
Copper concentration of the fruits sampled in the Awka market basket survey. Values represent mean Cu concentration ± standard deviation, n = 3. Codex indicates Codex food grade standard.^55^

**Figure 5:**
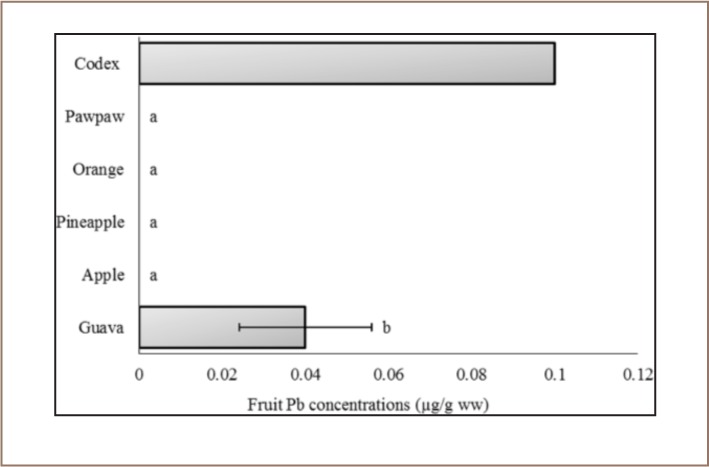
Lead concentration of the fruits sampled in the Awka market basket survey. Values represent mean Pb concentration ±standard deviation, n = 3. Mean values bearing different superscripts are significantly different (Kruskal Wallis with Bonferroni correction, P < 0.05). Codex indicates Codex food grade standard.^55^

The percentage moisture contents of all fruit samples examined were guava, 80.8%; apple, 84%; pineapple, 87%; orange, 87%; and paw paw, 85.5%. The data values were subsequently used to transform metal concentrations from dry weights to wet weights (*Figures 2–5*). The mean, minimum, and maximum total As, Hg, Cu and Pb concentrations (dw) for the independent fruit samples collected from Awka market are presented in Table 1, while the corresponding comparisons of the metal concentrations (ww) with Codex guideline levels on a wet weight basis are shown in Figures 2–5. The results show that As concentration was highest in pawpaw (96.84±0.00 μg/g dw) followed by pineapple (56.84±0.71 μg/g dw), while the least concentration was measured in orange (43.68±0.00 μg/g dw) (*Table 1*). Comparatively, in wet weight, the level of As was significantly higher (P < 0.05) than the Codex maximum level for As in fruits (*Figure 2*).

**Table 1:**
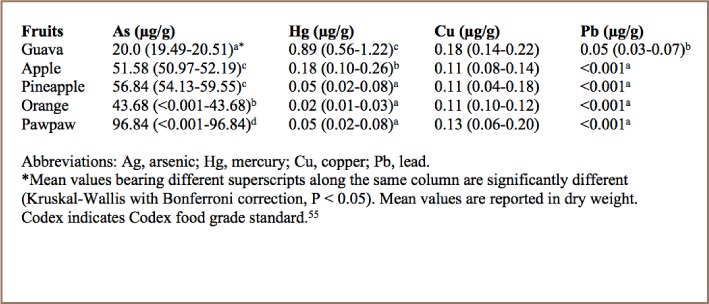
Average (minimum-maximum) Arsenic, Mercury, Copper and Lead Concentrations in Fruits Collected for the Awka Market Basket Survey

The Codex provides a guideline level for methyl Hg of 0.10 μg/g ww. Based on the supposition that methyl mercury is ~83% of total mercury, as presented in Table 1, the concentration of methyl Hg in our samples was calculated in wet weight and compared with the Codex guideline level, and guava and apple methyl Hg concentrations were ~6 and ~1 orders of magnitude higher than the Codex maximum guideline level, respectively (*Figure 3*).^54^ The amounts of Cu and Pb in the sampled fruits were within the Codex standards (*Figures 4 and 5*). Apple, pineapple, orange, and paw paw had Pb concentrations of <0.001 μg/g.

## Discussion

The present study investigated concentrations of As, Hg, Cu, and Pb in commonly consumed horticultural fruits to assess food quality and make recommendations based on an explicit understanding of human health risks. Metal contamination of edible fruits poses a potential health risk to both humans and animals. Awka South was selected for the current survey because of the open market system in the area, and because physical structural changes are rapidly taking place within its urban ecology, such as continuous road and building construction. These activities are, in many cases, not rigorously planned. Dust pollution and fog are common weather events that characterize the region, and markets are indiscriminately located along roads. Thus, monitoring of food contamination by metals and hazardous substances is necessary for the assessment and reduction of dietary exposures.^6^,^7^,^19^,^23^,^46^,^51^

The examined fruits had significantly higher total As concentrations (~32–166×) than the Codex maximum food grade level (0.5 μg/g ww). While fruit Cu and Pb concentrations were below the Codex standards, we found that Hg in guava and apple were 6.1 and 1.3 times higher than safe levels for consumption (0.1 μg/g ww), respectively.^55^ Fruits are capable of accumulating metals in their internal tissues, which are often species-specific and regulated by factors such as ecological setting and soil type.^56–59^ In some cases, surface contamination of fruits with hazardous substances are enabled by poor handling and processing.^6^ Our observation of relatively high As and Hg concentrations compared to Codex standards is particularly striking, and appears to indicate similar sources of contamination of these fruits. We postulate that surface/atmospheric deposition of soil and dust might be the primary mechanism of contamination.^20^,^60^ Awka has a high population density (~1283.2 inhabitants/km^2^ as of the 2006 census) and it is near the metropolitan area of Onitsha which has a much higher population density (2017 projected population of 1,318,660 at +9.03%/year) and industrial activities.^50^ There is scanty vegetation cover in Awka, which is subject to wind erosion and consequent agricultural contamination.^2–5^,^24^,^25^,^61^ Despite poor handling of fruits at the market where they are sold, wood processing is also a common activity within the vicinity and could contribute to discrete As environmental load and the resultant high concentrations measured in fruits. Arsenate is used in wood preservation and it is converted to more toxic arsenite by incineration of wood waste.^62^ With the indiscriminate waste disposal system in Awka, airborne pollutants may deposit on unprotected food displayed for sale. Previous studies have demonstrated that direct atmospheric deposition of As was the dominant pathway of contamination in leafy vegetables grown proximal to a wood preservation factory.^62^ Arsenic is a well known toxic element to humans with high levels of exposure.^40^ About 30%–95% of As is readily absorbed by humans when ingested, and children are more susceptible than adults to toxicity due to the lack of hepatic detoxification enzymes.^63^,^64^

Mercury is one of the most toxic elements among the studied trace metals and exposure to high levels could permanently damage the brain, kidneys and developing fetus.^65^ Concentrations of total Hg reported in fruit samples are expected to be mostly composed of methyl Hg.^54^,^66^ The effects of public health exposure to methyl mercury are critical. Epidemiological studies on daily oral ingestion of methyl Hg concentrations as low as 0.86 μg/g/day from fish alone in expectant mothers have been correlated with neurophysiological disorders in their children by 7 years of age.^67^ The highest Hg concentration in both dry weight and wet weight was found in guava and the lowest found in orange. Although atmospheric and surface deposition is speculated to be a primary vehicle of metal mobilization, we cannot rule out the possibility that these metals were also present in the fruit samples as a result of direct uptake from soil, which has been previously reported.^68^,^69^ Furthermore, we did not wash or peel the fruit samples prior to analysis, which may have raised the observed metal concentrations levels, as processing prior to analytical determination appears to reduce metal concentrations in some studies.^20^,^70^ The lower concentrations of Cu and Pb compared to the Codex guidelines indicates that fruits sold at the market may not have been contaminated with these metals at the time of sampling. There is lack of information on food trace metal contents in Awka and as this is the first market basket survey of fruits in this area, comparative analysis is difficult. However, the Hg concentrations measured in our work were higher than values (0.022–0.03 μg/g dw) reported in fruits collected from a Saudi Arabian market, but the fruit Cu and Pb concentrations (3.57–7.30 μg/g dw, and 2.62–6.70 μg/g dw, respectively) were ~33 and ~34× the highest Cu and Pb concentrations measured in our work, respectively.^15^ Market basket survey of some trace metals in fruits has been previously conducted in Owerri town, an area 117–121 km from Awka South, where a maximum Pb concentration of 4.33 μg/g dw was reported.^71^ The Pb level was ~21 orders of magnitude higher than the average Pb measured in our work. Interestingly, our present data on Cu and Pb concentrations in dry weight were ~11× and ~2× the maximum concentrations (0.02 and 0.13 μg/g, respectively) measured in fruits purchased from markets in the most densely populated and industrially active Nigerian city of Lagos.^72^ The observed difference in total trace metal concentrations between the published studies indicates variation in metal contaminations in response to spatial characteristics and other factors such as pollutant mobilization, deposition and or contamination rates. Although Cu is an essential element needed for biological mechanisms in both humans and animals, the measured concentrations of Pb in the examined fruits are of concern due to possible bioaccumulation and dietary toxicity.^73^ The International Agency for Research on Cancer has classified inorganic Pb compounds as probably carcinogenic to humans (Group 2A).^74^ Lead toxicity has numerous negative human health effects. For example, exposure to Pb in pre- and postnatal development periods causes delayed or reduced neurological and sexual development.^75^ Children are more susceptible than adults to the effects of Pb toxicity.^76^

## Conclusions

The present study shows that the concentrations of common metalloids and trace metals in fruits is varied and highlights the potential impact of anthropogenic activities on metal mobility, deposition, and food contamination. The elevated As and Hg concentrations measured in the fruits indicate potential dietary exposure to metals, and may be of great public health concern. Although Pb concentrations were lower than Codex standards, there is a need to investigate whether these low exposures may contribute to risks of adverse health effects given recent studies indicating health effects at relatively low concentrations. This is a pilot-based screening; larger-scale monitoring is necessary to assess fruits not included in the current survey and correlate contamination levels with metal concentrations in farm soil and atmospheric dust. Food intake assessment methods based on a more comprehensive determination of food types and consumption patterns of the local population need to be implemented and individual consumption data generated to facilitate the risk assessments of metals. Although the present investigation did not examine the effects of adulterants on imported apples, the screening system should be expanded to monitor and control their use on fruits, as there is evidence of adverse health effects associated with adulterants in the food industry.^77^ Further studies are needed to continue to improve the database on food metal contents for comparative studies. Moreover, an analytical approach for As and Hg speciation in fruits is necessary for effective food safety assessment. The development of a more robust risk communication framework is needed for better information on specific dietary exposures and food contamination.

## References

[1] The importance of food quality and safety for developing countries [Internet]. Committee on World Food Security: 25th Session; 1999 May 31 – June 3; Rome Rome: Food and Agriculture Organisation; 1999 Apr [cited 2016 Dec 11] 13 p. Available from: ftp://ftp.fao.org/unfao/bodies/cfs/cfs25/X1845e.doc

[2] Ezeonyejiaku CD, Obiakor MO. Metal enrichment in water and fish in a semi-urban Nigerian lake, and their associated risks. *Afr J Aquat Sci* [Internet]. 2016 [cited 2017 Jul 12]; 41 1: 41– 9. Available from: http://www.tandfonline.com/doi/abs/10.2989/16085914.2015.1136803 Subscription required to view.

[3] Obiakor MO, Okonkwo JC, Ezeonyejiaku CD. Trace element contamination in tropical endemic fish: factorial effect interactions and in situ quantitative risk assessment. *J Environ Occup Sci* [Internet]. 2015 Jan-Mar [cited 2017 Jul 12]; 4 1: 10– 21. Available from: http://www.ejmanager.com/fulltextpdf.php?mno=173680

[4] Obiakor MO, Okonkwo JC, Ezeonyejiaku CD, Ezenwelu CO. Physicochemical and heavy metal distribution in freshwater column: season-location interaction effects and public health risk. *J Life Sci Biomed* [Internet]. 2013 [cited 2017 Jul 12]; 3 4: 308– 17. Available from: http://jlsb.science-line.com/attachments/article/23/J.%20Life%20Sci.%20Biomed.%203(4)%20308-317,%202013.pdf

[5] Obiakor MO, Okonkwo JC, Ezeonyejiaku CD, Okonkwo CN. Polycyclic aromatic hydrocarbons (pahs) in freshwater media: factorial effects and human dietary exposure risk assessment. *Resour Environ* [Internet]. 2014 [cited 2017 Jul 12]; 4 6: 247– 59. Available from: http://article.sapub.org/10.5923.j.re.20140406.01.html

[6] Food safety: what you should know [Internet]. Geneva, Switzerland: World Health Organization; 2015 4 7 [cited 2017 Jan 10]. [about 25 screens]. Available from: http://www.searo.who.int/entity/world_health_day/2015/whd-what-you-should-know/en/

[7] Andras P, Turisovs I, Krnac J, Dirner V, Volekov-Lalinska B, Buccheri G, Jelen S. Hazards of heavy metal contamination at L'ubietová Cu-Deposit (Slovakia). *Procedia Environ Sci* [Internet]. 2012 [cited 2017 Jul 12]; 14: 3– 21. Available from: http://www.sciencedirect.com/science/article/pii/S1878029612004720

[8] Hayes RB. The carcinogenicity of metals in humans. Cancer Causes Control [Internet]. 1997 5 [cited 2017 Jul 12]; 8 3: 371– 85. Available from: http://link.springer.com/article/10.1023%2FA%3A1018457305212 Subscription required to view. 10.1023/a:1018457305212 9498900

[9] Jarup L. Hazards of heavy metal contamination. *Brit Med Bull* [Internet]. 2003 [cited 2017 Jul 12]; 68 1: 167– 82. Available from: https://academic.oup.com/bmb/article/68/1/167/421303/Hazards-of-heavy-metal-contamination?searchresult=1 10.1093/bmb/ldg032 14757716

[10] Khan S, Cao Q, Zheng YM, Huang YZ, Zhu YG. Health risks of heavy metals in contaminated soils and food crops irrigated with wastewater in Beijing, China. *Environ Pollut* [Internet]. 2008 4 [cited 2017 Jul 12]; 152 3: 686– 92. Available from: http://www.sciencedirect.com/science/article/pii/S0269749107003351 Subscription required to view. 10.1016/j.envpol.2007.06.056 17720286

[11] Wuana RA, Okieimen FE. Heavy metals in contaminated soils: a review of sources, chemistry, risks and best available strategies for remediation. *ISRN Ecol* [Internet]. 2011 [cited 2017 Jul 12]; 2011: 1– 20. Available from: https://www.hindawi.com/journals/isrn/2011/402647/

[12] Shibata T, Meng C, Umoren J, West H. Risk assessment of arsenic in rice cereal and other dietary sources for infants and toddlers in the U.S. *Int J Environ Res Public Health* [Internet]. 2016 4 [cited 2017 Jul 12]; 13 4: 361 Available from: http://www.ncbi.nlm.nih.gov/pmc/articles/PMC4847023/ 10.3390/ijerph13040361 PMC4847023 27023581

[13] Nriagu JO. Global metal pollution: poisoning the biosphere? *Environ: Sci Policy Sustain Dev* [Internet]. 1990 [cited 2017 Jul 12]; 32 7: 7– 33. Available from: http://www.tandfonline.com/doi/abs/10.1080/00139157.1990.9929037 Subscription required to view.

[14] Nriagu JO, Pacyna JM. Quantitative assessment of worldwide contamination of air, water and soils by trace metals. *Nat* [Internet]. 1988 5 12 [cited 2017 Jul 12]; 333: 134– 9. Available from: http://www.nature.com/nature/journal/v333/n6169/abs/333134a0.html Subscription required to view. 10.1038/333134a0 3285219

[15] Ali MH, Al-Qahtani KM. Assessment of some heavy metals in vegetables, cereals and fruits in Saudi Arabian markets. *Egypt J Aquat Res* [Internet]. 2012 [cited 2017 Jul 12]; 38 1: 31– 7. Available from: http://www.sciencedirect.com/science/article/pii/S1687428512000039

[16] Dolan LC, Matulka RA, Burdock GA. Naturally occurring food toxins. *Toxins* [Internet]. 2010 [cited 2017 Jul 13]; 2 9: 2289– 332. Available from: http://www.mdpi.com/2072-6651/2/9/2289 10.3390/toxins2092289 PMC3153292 22069686

[17] Shaheen N, Irfan NM, Khan IN, Islam S, Islam MS, Ahmed MK. Presence of heavy metals in fruits and vegetables: health risk implications in Bangladesh. *Chemosphere [*Internet]. 2016 6 [cited 2017 Jul 13]; 152: 431– 8. Available from: http://www.sciencedirect.com/science/article/pii/S0045653516302120 Subscription required to view. 10.1016/j.chemosphere.2016.02.060 27003365

[18] Taghipour H, Mosaferi M. Heavy metals in the vegetables collected from production sites. *Health Promot Perspect* [Internet]. 2013 [cited 2017 Jul 13]; 3 2: 185– 93. Available from: http://www.ncbi.nlm.nih.gov/pmc/articles/PMC3963666/ 10.5681/hpp.2013.022 PMC3963666 24688968

[19] Yusuf AA, Arowolo TA, Bamgbose O. Cadmium, copper and nickel levels in vegetables from industrial and residential areas of Lagos City, Nigeria. *Food Chem Toxicol* [Internet]. 2003 3 [cited 2017 Jul 13]; 41 3: 375– 8. Available from: http://www.sciencedirect.com/science/article/pii/S0278691502002235 Subscription required to view. 10.1016/s0278-6915(02)00223-5 12504169

[20] Norton G, Deacon C, Mestrot A, Feldmann J, Jenkins P, Baskaran C, Meharg AA. Arsenic speciation and localization in horticultural produce grown in a historically impacted mining region. *Environ Sci Technol* [Internet]. 2013 [cited 2017 Jul 13]; 47 12: 6164– 72. Available from: http://pubs.acs.org/doi/abs/10.1021/es400720r Subscription required to view. 10.1021/es400720r 23688252

[21] Voutsa D, Grimanis A, Samara C. Trace elements in vegetables grown in an industrial area in relation to soil and air particulate matter. *Environ Pollut* [Internet]. 1996 [cited 2017 Jul 13]; 94 3: 325– 35. Available from: http://www.sciencedirect.com/science/article/pii/S0269749196000887 Subscription reqired to view. 10.1016/s0269-7491(96)00088-7 15093493

[22] Kiende JI, Kawaka F, Orinda G, Okemo P. Assessment of heavy metal concentrations in urban grown vegetables in Thika Town, Kenya. *Afr J Food Sci* [Internet]. 2012 2 [cited 2017 Jul 13]; 6 3: 41– 6. Available from: http://ir-library.ku.ac.ke/handle/123456789/5819

[23] Zahir E, Naqvi II, Uddin SM. Market basket survey of selected metals in fruits from Karachi City (Pakistan). *J Basic Appl Sci* [Internet]. 2009 [cited 2017 Jul 13]; 5 2: 47– 52. Available from: https://www.researchgate.net/profile/Erum_Zahir/publication/228355837_Market_Basket_Survey_of_selected_metals_in_fruits_from_Krachi_city_Pakistan/links/02e7e51a31f52232de000000.pdf

[24] Ezeonyejiaku CD, Nwuba LA, Obiakor MO, Okonkwo CN. Bioaccumulation of heavy metals in fish sourced from environmentally stressed axis of River Niger: threat to ecosystem and public health. *Int J Environ Prot Policy* [Internet]. 2014 [cited 2017 Jul 13]; 2 4: 126– 31. Available from: http://article.sciencepublishinggroup.com/pdf/10.11648.j.ijepp.20140204.11.pdf

[25] Ezeonyejiaku CD, Obiakor MO. Physicochemical and heavy metal profile of surface water, anthropogenic activities, and community health implications. *J Environ Conserv Res* [Internet]. 2013 [cited 2017 Jul 13]; 1 2: 40– 8. Available from: https://www.researchgate.net/publication/256383038_Physicochemical_and_Heavy_Metal_Profile_of_Surface_Water_Anthropogenic_Activities_and_Community_Health_Implications

[26] Chen CY, Borsuk ME, Bugge DM, Hollweg T, Balcom PH, Ward DM, Williams J, Mason RP. Benthic and pelagic pathways of methylmercury bioaccumulation in estuarine food webs of the Northeast United States. *PLoS ONE* [Internet]. 2014 [cited 2017 Jul 13]; 9 2: e89305 Available from: http://journals.plos.org/plosone/article?id=10.1371/journal.pone.0089305 10.1371/journal.pone.0089305 PMC3928433 24558491

[27] Chen CY, Dionne M, Mayes BM, Ward DM, Sturup S, Jackson BP. Mercury bioavailability and bioaccumulation in estuarine food webs in the Gulf of Maine. *Environ Sci Technol* [Internet]. 2009 [cited 2017 Jul 13]; 43 6: 1804– 10. Available from: http://pubs.acs.org/doi/abs/10.1021/es8017122 Subscription required to view. 10.1021/es8017122 PMC2670462 19368175

[28] Chen CY, Stemberger RS, Klaue B, Blum JD, Pickhardt PC, Folt CL. Accumulation of heavy metals in food web components across a gradient of lakes. *Limnol Oceanogr* [Internet]. 2000 11 [cited 2017 Jul 13]; 45 7: 1525– 36. Available from: http://onlinelibrary.wiley.com/doi/10.4319/lo.2000.45.7.1525/abstract;jsessionid=34428389F02EF335F9150E64E82E52C2.f04t03

[29] Chen CY, Ward DM, Williams JJ, Fisher NS. Metal bioaccumulation by estuarine food webs in New England, USA. *J Mar Sci Eng* [Internet]. 2016 [cited 2017 Jul 13]; 4 2: 1– 15. Available from: http://www.mdpi.com/2077-1312/4/2/41/htm 10.3390/jmse4020041 PMC5455787 28580179

[30] Qiu Y-W. Bioaccumulation of heavy metals both in wild and mariculture food chains in Daya Bay, South China. *Estuar Coast Shelf Sci* [Internet]. 2015 [cited 2017 Jul 13]; 163 Part B: 7– 14. Available from: http://www.sciencedirect.com/science/article/pii/S0272771415001924 Subscription required to view.

[31] Zhang G, Pan Z, Hou X, Wang X, Li X. Distribution and bioaccumulation of heavy metals in food web of Nansi Lake, China. *Environ Earth Sci* [Internet]. 2015 3 [cited 2017 Jul 13]; 73 5: 2429– 39. Available from: http://link.springer.com/article/10.1007%2Fs12665-014-3592-z Subscription required to view.

[32] Turkdogan MK, Kilicel F, Kara K, Tuncer I, Uygan I. Heavy metals in soil, vegetables and fruits in the endemic upper gastrointestinal cancer region of Turkey. *Environ Toxicol Pharm* [Internet]. 2003 4 [cited 2017 Jul 13]; 13 3: 175– 9. Available from: http://www.sciencedirect.com/science/article/pii/S1382668902001564 Subscription required to view. 10.1016/S1382-6689(02)00156-4 21782652

[33] Yuan W, Yang N, Li X. Advances in understanding how heavy metal pollution triggers gastric cancer. *BioMed Res Int* [Internet]. 2016 [cited 2017 Jul 13]; 2016: 1– 10. Available from: https://www.hindawi.com/journals/bmri/2016/7825432/ 10.1155/2016/7825432 PMC5075591 27803929

[34] Tchounwou PB, Yedjou CG, Patlolla AK, Sutton DJ. Heavy metal toxicity and the environment. : Luch A, editor Molecular, clinical and environmental toxicology [Internet]. Vol 3: environmental toxicology. Basel, Switzerland: Springer Basel; 2012 [cited 2017 Jul 13]. p. 133– 64. Available from: http://link.springer.com/chapter/10.1007%2F978-3-7643-8340-4_6. Subscription required to view. 10.1007/978-3-7643-8340-4_6 PMC4144270 22945569

[35] Wilson SC, Tighe M, Paterson E, Ashley PM. Food crop accumulation and bioavailability assessment for antimony (Sb) compared with arsenic (As) in contaminated soils. *Environ Sci Pollut Res* [Internet]. 2014 10 [cited 2017 Jul 13]; 21 20: 11671– 81. Available from: http://link.springer.com/article/10.1007%2Fs11356-014-2577-5. Subscription required to view. 10.1007/s11356-014-2577-5 24499989

[36] Azcue M, Nriagu JO. Arsenic: historical perspective. : Nriagu JO, editor Arsenic in the environment, part 1: cycling and characterisation. Toronto: John Wiley & Sons; 1994 4 [cited 2017 Jul 13] 448 p. Available from: http://au.wiley.com/WileyCDA/WileyTitle/productCd-0471579297.html Subscription required to view.

[37] Hong Y-S, Song K-H, Chung J-Y. Health effects of chronic arsenic exposure. *J Prev Med Public Health* [Internet]. 2014 9 [cited 2017 Jul 12]; 47 5: 245– 52. Available from: http://www.ncbi.nlm.nih.gov/pmc/articles/PMC4186552/ 10.3961/jpmph.14.035 PMC4186552 25284195

[38] Hughes MF, Beck BD, Chen Y, Lewis AS, Thomas DJ. Arsenic exposure and toxicology: a historical perspective. *Toxicol Sci* [Internet]. 2011 [cited 2017 Jul 12]; 123 2: 305– 32. Available from: https://academic.oup.com/toxsci/article-lookup/doi/10.1093/toxsci/kfr184 10.1093/toxsci/kfr184 PMC3179678 21750349

[39] Kapaj S, Peterson H, Liber K, Bhattacharya P. Human health effects from chronic arsenic poisoning-a review. *J Environ Sci Health A Tox Hazard Subst Environ Eng* [Internet]. 2006 [cited 2017 Jul 12]; 41 10: 2399– 428. Available from: http://www.tandfonline.com/doi/full/10.1080/10934520600873571 Subscription required to view. 10.1080/10934520600873571 17018421

[40] Arsenic toxicity: what are the physiologic effects of arsenic exposure? Atlanta, Georgia: Agency for Toxic Substances and Disease Registry; 2009 10 1 [updated 2011 Oct 1; cited 2016 Mar 23]. p 42– 59. Available from: https://www.atsdr.cdc.gov/csem/arsenic/docs/arsenic.pdf

[41] Some drinking-water disinfectants and contaminants, including arsenic [Internet]. International Agency for Research on Cancer Working Group on the Evaluation of Carcinogenic Risks to Humans; 2002 Oct 15–22; Lyon, France Geneva, Switzerland: World Health Organization; 2004 [cited 2017 Jul 13] 526 p. (vol. 84) Available from: http://monographs.iarc.fr/ENG/Monographs/vol84/index.php

[42] Liu CP, Luo CL, Gao Y, Li FB, Lin LW, Wu CA, Li XD. Arsenic contamination and potential health risk implications at an abandoned tungsten mine, southern China. *Environ Pollut* [Internet]. 2010 3 [cited 2017 Jul 13]; 158 3: 820– 6. Available from: http://www.sciencedirect.com/science/article/pii/S0269749109004813 Subscription required to view. 10.1016/j.envpol.2009.09.029 19910093

[43] Munoz O, Diaz OP, Leyton I, Nunez N, Devesa V, Suner MA, Velez C, Montoro R. Vegetables collected in the cultivated Andean area of northern Chile: total and inorganic arsenic contents in raw vegetables. *J Agric Food Chem* [Internet]. 2002 [cited 2017 Jul 13]; 50 3: 642– 7. Available from: http://pubs.acs.org/doi/abs/10.1021/jf011027k. Subscription required to view. 10.1021/jf011027k 11804542

[44] Slejkovec Z, van Elteren JT, Glass H-J, Jeran Z, Jacimovic R. Speciation analysis to unravel the soil-to-plant transfer in highly arsenic-contaminated areas in Cornwall (UK). *Int J Environ Anal Chem* [Internet]. 2010 [cited 2017 Jul 13]; 90 10: 784– 96. Available from: http://www.tandfonline.com/doi/abs/10.1080/03067310902977542 Subscription required to view.

[45] Williams PN, Islam MR, Adomako EE, Raab A, Hossain SA, Zhu YG, Feldmann J, Meharg AA. Increase in rice grain arsenic for regions of Bangladesh irrigating paddies with elevated arsenic in groundwaters. *Environ Sci Technol* [Internet]. 2006 [cited 2017 Jul 13]; 40 16: 4903– 8. D Available from: http://pubs.acs.org/doi/abs/10.1021/es060222i Subscription required to view. 10.1021/es060222i 16955884

[46] Scientific opinion on arsenic in food. EFSA J [Internet]. 2009 10 [cited 2017 Jul 13]; 7 10: 1351 [199 p]. Available from: http://onlinelibrary.wiley.com/doi/10.2903/j.efsa.2009.1351/full

[47] Fatiregun AA, Oyebade OA, Oladokun L. Investigation of an outbreak of food poisoning in a resource-limited setting. *Trop J Health Sci* [Internet]. 2010 [cited 2017 Jul 13]; 17 1 Available from: http://www.ajol.info/index.php/tjhc/article/view/52816 Subscription required to view.

[48] Okonkwo EE, Ibeanu AM. Nigeria's archaeological heritage. SAGE Open [Internet]. 2016 Apr-Jun [cited 2017 Jul 13]; 6 2: 1– 7. Available from: http://journals.sagepub.com/doi/abs/10.1177/2158244016651111

[49] Onyido AE, Zeibe CC, Okonkwo NJ, Ezugbo-Nwobi IK, Egbuche CM, Udemezue IO, Ezeanya LC. Damage caused by the bean bruchid, Callosobruchus Maculatus (Fabricius) on different legume seeds on sale in Awka and Onitsha markets, Anambra State, Southeastern Nigeria. *Afr Res Rev* [Internet]. 2011 [cited 2017 Jul 13]; 5 4: 116– 23. Available from: http://www.ajol.info/index.php/afrrev/article/view/69264

[50] NPC. National and state population. Abuja: National Population Commission (NPC) of Nigeria, 2017 Available from: http://www.population.gov.ng/index.php/censuses

[51] Norton G, Deacon C, Mestrot A, Meharg A. Total cadmium, copper, lead and zinc in fruit and vegetables grown in the UK [Internet]. London: Food Standards Agency; 2012 1 6 [cited 2016 Dec 22] 104 p. Report No.: FS241003. Available from: https://www.food.gov.uk/sites/default/files/research-multielement-report-arsenic-fruit-veg.pdf

[52] Shapiro SS, Wilk MB. An analysis of variance test for normality (complete samples). *Biometrika* [Internet]. 1965 12 1 [cited 2017 Jul 13]; 52 3–4: 591– 611. Available from: http://biomet.oxfordjournals.org/content/52/3-4/591.short Subscription required to view.

[53] Conover WJ. Practical nonparametric statistics. 3rd ed New York, NY: John Wiley & Sons; 1999 12 14 592 p.

[54] Kannan K, Smith R.G. J, Lee RF, Windom HL, Heitmuller PT, Macauley JM, Summers JK. Distribution of total mercury and methyl mercury in water, sediment, and fish from South Florida Estuaries. *Arch Environ Contam Toxicol* [Internet]. 1998 2 [cited 2017 Jul 13]; 34 2: 109– 18. Available from: 10.1007/s002449900294 Subscription required to view. 9469852

[55] Codex general standard for contaminants and toxins in food and feed: CODEX STAN 193-1995 [Internet]. Rome, Italy: Food and Agriculture Organization; 1995 [cited 2017 Jul 13[ 44 p Available from: http://www.fao.org/fileadmin/user_upload/livestockgov/documents/1_CXS_193e.pdf

[56] Chang CY, Yu HY, Chen JJ, Li FB, Zhang HH, Liu CP. Accumulation of heavy metals in leaf vegetables from agricultural soils and associated potential health risks in the Pearl River Delta, South China. Environ Monit Assess [Internet]. 2014 Mar [cited 2017 Jul 13]; 186 3: 1547– 60. Available from: http://link.springer.com/article/10.1007/s10661-013-3472-0 Subscription required to view. 24185814 10.1007/s10661-013-3472-0 PMC3902199

[57] McBride MB. Arsenic and lead uptake by vegetable crops grown on historically contaminated orchard soils. *Appl Environ Soil Sci* [Internet]. 2013 [cited 2017 Jul 13]; 2013: 1– 8. Available from: https://www.hindawi.com/journals/aess/2013/283472/ 10.1155/2013/283472 PMC4776765 26949393

[58] McBride MB, Shayler HA, Russell-Anelli JM, Spliethoff HM, Marquez-Bravo LG. Arsenic and lead uptake by vegetable crops grown on an old orchard site amended with compost. *Water Air Soil Pollut* [Internet]. 2015 8 [cited 2017 Jul 13]; 226 8: 265 Available from: http://link.springer.com/article/10.1007%2Fs11270-015-2529-9 10.1007/s11270-015-2529-9 PMC4755492 26900187

[59] Bhatti SM, Anderson CWN, Stewart RB, Robinson BH. Risk assessment of vegetables irrigated with arsenic-contaminated water. *Environ Sci: Process Impacts* [Internet]. 2013 [cited 2017 Jul 13]; 15 10: 1866– 75. Available from: http://pubs.rsc.org/en/Content/ArticleLanding/2013/EM/c3em00218g#!divAbstract Subscription required to view. 10.1039/c3em00218g 23934025

[60] Bussinow M, Sarapatka B, Dlapa P. Effect of old mining activities on nutrient and toxic elements concentration in the biomass of Norway spruce (Picea abies L. Karst.) and European Birch (Betula pendula L.). *Int J Environ Pollut* [Internet]. 2008 [cited 2017 Jul 13]; 33 2–3: 235– 47. Available from: http://www.inderscienceonline.com/doi/abs/10.1504/IJEP.2008.019396 Subscription required to view.

[61] Obiakor MO, Ezeonyejiaku CD, Mogbo TC. Effects of vegetated and synthetic (impervious) surfaces on the microclimate of urban area. *J Appl Sci* Environ Manag [Internet]. 2012 3 [cited 2017 Jul 13]; 16 1: 85– 94. Available from: http://www.bioline.org.br/pdf?ja12014

[62] Larsen EH, Moseholm L, Nielsen MM. Atmospheric deposition of trace elements around point sources and human health risk assessment. II: Uptake of arsenic and chromium by vegetables grown near a wood preservation factory. *Sci Total Environ* [Internet]. 1992 9 25 [cited 2017 Jul 13]; 126 3: 263– 75. Available from: http://www.sciencedirect.com/science/article/pii/0048969792902013 Subscription required to view. 10.1016/0048-9697(92)90201-3 1439755

[63] Toxicological profile for arsenic [Internet]. Atlanta, GA: Agency for Toxic Substances and Disease Registry; 2007 8 [updated 2005 Sep; cited 2016 Mar 23] 559 p. Available from: http://www.atsdr.cdc.gov/toxprofiles/tp2.pdf 37184170

[64] Nordberg GF, Fowler BA, Nordberg M. Handbook on the toxicology of metals. 4th ed Cambridge, Massachusetts: Academic Press; 2014 8 29 1542 p.

[65] Castro-Gonzalez MI, Mendez-Armenta M. Heavy metals: implications associated to fish consumption. *Environ Toxicol Pharm* [Internet]. 2008 11 [cited 2017 Jul 13]; 26 3: 263– 71. Available from: http://www.sciencedirect.com/science/article/pii/S1382668908000914 Subscription required to view. 10.1016/j.etap.2008.06.001 21791373

[66] B Commission regulation (EC) No 1881/2006: of 19 December 2006: setting maximum levels for certain contaminants in foodstuffs. Official Journal of the European Union Legislation 364 (Aug. 7, 2015).

[67] Methylmercury (MeHg): CASRN 22967-92-6 [Internet]. Washington, D.C.: United States Environmental Protection Agency; 1988 [updated 2001 Aug 27; cited 2017 Mar 28] 43 p. Available from: https://cfpub.epa.gov/ncea/iris/iris_documents/documents/subst/0073_summary.pdf

[68] Samsoe-Petersen L, Larsen EH, Larsen PB, Bruun P. Uptake of trace elements and PAHs by fruit and vegetables from contaminated soils. Environ Sci Technol [Internet]. 2002 [cited 2017 Jul 13]; 36 14: 3057– 63. Available from: 10.1021/es015691t Subscription required to view. 12141482

[69] Roba C, Rosu C, Pistea I, Ozunu A, Baciu C. Heavy metal content in vegetables and fruits cultivated in Baia Mare mining area (Romania) and health risk assessment. *Environ Sci Pollut Res* [Internet]. 2016 4 [cited 2017 Jul 13]; 23 7: 6062– 73. Available from: 10.1007/s11356-015-4799-6 Subscription required to view. 26062461

[70] Norton GJ, Deacon CM, Mestrot A, Feldmann J, Jenkins P, Baskaran C, Meharg AA. Cadmium and lead in vegetable and fruit produce selected from specific regional areas of the UK. *Sci Total Environ* [Internet]. 2015 11 15 [cited 2017 Jul 13]; 533: 520– 7. Available from: http://www.sciencedirect.com/science/article/pii/S0048969715303314 Subscription required to view. 10.1016/j.scitotenv.2015.06.130 26188403

[71] Orisakwe OE, Nduka JK, Amadi CN, Dike DO, Bede O. Heavy metals health risk assessment for population via consumption of food crops and fruits in Owerri, South Eastern, Nigeria. *Chem Central J* [Internet]. 2012 [cited 2017 Jul 13]; 6 77: 1– 7. Available from: http://ccj.springeropen.com/articles/10.1186/1752-153X-6-77 10.1186/1752-153X-6-77 PMC3567425 22853175

[72] Sobukola OP, Adeniran OM, Odedairo AA, Kajihausa OE. Heavy metal levels of some fruits and leafy vegetables from selected markets in Lagos, Nigeria. Afr J Food Sci [Internet]. 2010 6 [cited 2017 Jul 13]; 4 2: 389– 93. Available from: http://www.academicjournals.org/article/article1380725945_Sobukola%20et%20al.pdf

[73] Bost M, Houdart S, Oberli M, Kalonji E, Huneau J-F, Margaritis I. Dietary copper and human health: current evidence and unresolved issues. *J Trace Elements Med Biol* [Internet]. 2016 5 [cited 2017 Jul 13]; 35: 107– 15. Available from: http://www.sciencedirect.com/science/article/pii/S0946672X16300207 10.1016/j.jtemb.2016.02.006 27049134

[74] Inorganic and organic lead compounds [Internet]. International Agency for Research on Cancer Working Group on the Evaluation of Carcinogenic Risks to Humans; 2004 Feb 10–17; Lyon, France Geneva, Switzerland: World Health Organization; 2006 [cited 2016 May 19] 529 p. (vol. 87) Available from: http://monographs.iarc.fr/ENG/Monographs/vol87/mono87.pdf

[75] Toxicological profile for lead [Internet]. Atlanta, GA: Agency for Toxic Substances and Disease Registry; 2007 8 [updated 2005 Sep; cited 2016 Jan 21] 582 p. Available from: http://www.atsdr.cdc.gov/toxprofiles/tp13.pdf 24049859

[76] Jarup L. Hazards of heavy metal contamination. *Br Med Bull* [Internet]. 2003 [cited 2017 Jul 13]; 68 1: 167– 82. Available from: 10.1093/bmb/ldg032 14757716

[77] Majed N, Real IH, Akter M, Azam HM. Food adulteration and bio-magnification of environmental contaminants: a comprehensive risk framework for Bangladesh. *Front Environ Sci* [Internet]. 2016 5 18 [cited 2017 Jul 13]; 4 34: 1– 21. Available from: http://journal.frontiersin.org/article/10.3389/fenvs.2016.00034

